# Molecular study of the perforin gene in familial hematological malignancies

**DOI:** 10.1186/1897-4287-9-9

**Published:** 2011-09-21

**Authors:** Rim El Abed, Violaine Bourdon, Ilia Voskoboinik, Halima Omri, Yosra Ben Youssef, Mohamed Adnene Laatiri, Laetitia Huiart, François Eisinger, Laetitia Rabayrol, Marc Frenay, Paul Gesta, Liliane Demange, Hélène Dreyfus, Valérie Bonadona, Catherine Dugast, Hélène Zattara, Laurence Faivre, Monia Zaier, Saloua Yacoub Jemni, Testsuro Noguchi, Hagay Sobol, Zohra Soua

**Affiliations:** 1 UR Biologie moléculaire des leucémies et lymphomes - Faculté de Médecine de Sousse, Université de Sousse, Avenue Hamed Karoui; Sousse 4002, Tunisia; 2 Département d'Oncologie Génétique, de Prévention et Dépistage, Institut Paoli-Calmettes, 232 Boulevard Sainte Marguerite, Marseille, 13009, France; 3 Cancer Cell Death Laboratory, Cancer Immunology Program, Peter MacCallum Cancer Centre, St Andrew's Place, East Melbourne, VIC 3002, Australia; 4 Service d'Hématologie Clinique, CHU F. Hached, Avenue Ibn El Jazzar, Sousse, 4000, Tunisia; 5 Centre Antoine Lacassagne, 33 Avenue de Valombrose, Nice, 06189 Cedex 2, France; 6 CHG Niort, 40 avenue Charles de Gaulle, Niort, 79021, France; 7 Polyclinique de Courlancy, 38 Rue de Courlancy, Reims, 51100, France; 8 Institut Ste Catherine, 1750 chemin du Lavarin, Avignon, 84000, France; 9 Unité de génétique Epidémiologique, Centre Léon Bérard Lyon, 28 rue Laennec, Lyon, 69373 Cedex 08, France; 10 Centre Eugène-Marquis, Rue de la Bataille Flandre-Dunkerque, Rennes 35042, France; 11 Département de Génétique, Hôpital de la Timone, 264 Rue Saint-Pierre, Marseille, 13385, France; 12 Centre de Génétique, Hôpital d'Enfants, CHU de Dijon, 1 boulevard Jeanne d'Arc, Dijon, 21079, France; 13 Unité d'oncogénétique, Centre Georges François Leclerc, 1 rue du Professeur Marion, Dijon, 21034 Cedex, France; 14 Centre régional de transfusion sanguine de Sousse, CHU F. Hached, Avenue Ibn El Jazzar, Sousse, 4000, Tunisia; 15 Université d'Aix Marseille II, Avenue de Luminy, Marseille, 13009, France

**Keywords:** *PRF1*, germline mutation, hematological familial malignancies

## Abstract

Perforin gene (*PRF1*) mutations have been identified in some patients diagnosed with the familial form of hemophagocytic lymphohistiocytosis (HLH) and in patients with lymphoma. The aim of the present study was to determine whether patients with a familial aggregation of hematological malignancies harbor germline perforin gene mutations. For this purpose, 81 unrelated families from Tunisia and France with aggregated hematological malignancies were investigated. The variants detected in the *PRF1 *coding region amounted to 3.7% (3/81). Two of the three variants identified were previously described: the p.Ala91Val pathogenic mutation and the p.Asn252Ser polymorphism. A new p.Ala 211Val missense substitution was identified in two related Tunisian patients. In order to assess the pathogenicity of this new variation, bioinformatic tools were used to predict its effects on the perforin protein structure and at the mRNA level. The segregation of the mutant allele was studied in the family of interest and a control population was screened. The fact that this variant was not found to occur in 200 control chromosomes suggests that it may be pathogenic. However, overexpression of mutated *PRF1 *in rat basophilic leukemia cells did not affect the lytic function of perforin differently from the wild type protein.

## Background

Perforin is a Ca^2+ ^dependent pore forming protein stored as an active protein in specialized secretory lysosomes (known as lytic granules) of Cytotoxic T lymphocyte (CTL) and Natural Killer cells (NK). Upon recognition of the target cells, lytic granules polarize and release their contents at the immunologic synapse, which triggers apoptosis [1,2]. Cytotoxic granules also contain a group of serine proteases called granzymes in a proteoglycan matrix [3,4]. Perforin is the only molecule that is able to deliver granzymes into the target cell.

Perforin is encoded by *PRF1*, a highly conserved gene, which is crucial to the function of the granzymes involved in triggering caspase dependent and caspase independent target cell death after the formation of an immunological synapse [5]. Perforin-mediated cellular cytotoxicity is a highly preserved mechanism responsible for killing virus-infected and neoplastic cells.

*PRF1 *mutations were first described in familial hemophagocytic lymphohistiocytosis (FHL) [6,7]. These mutations include nonsense, frameshift and missense mutations disrupting perforin activity [8-15]. FHL is a life threatening disease usually occurring in childhood, which is associated with profound immune derangement and characterized by impaired T-cell and NK cell granule-mediated cytotoxic activity. The fact that these mutations were described in homozygous and compound heterozygous states suggests that autosomal recessive transmission processes are involved. Patients with FHL caused by biallelic perforin mutations are severely immunocompromised [7,16].

Inherited *PRF1 *mutations were subsequently described in various types of lymphomas [17-19], which suggests that PRF1 protein is involved in the immune surveillance mechanisms preventing tumor growth and/or development.

Escape from immune surveillance is thought to be the main mechanism possibly explaining the role of some predisposing genetic mutations in the development of leukemia and lymphoma[20-23].

The key role of perforin in immune surveillance has been extensively investigated using perforin knockout (*PRF1*-KO) mice, which show high sensitivity to several viral infections. These *PRF1*-KO mice develop spontaneous and aggressive disseminated B-cell lymphoma, and fail to efficiently reject many transplanted tumors [24-27].

Nevertheless, some previous studies have shown that hematological neoplasms can be transmitted vertically, which suggests that a predisposition may be caused by an inherited genetic factor with incomplete penetrance and pleitropic effects [28,29]. In view of these experimental findings, we tested the hypothesis that some patients with familial hematological malignancies might harbor perforin gene mutations. *PRF1 *germline mutations were therefore analyzed in a panel of families with aggregated hematological malignancies with or without solid tumors.

## Methods

### Patients and control population

The entire *PRF1 *coding region was sequenced in 89 patients belonging to 81 independent families: 6 Tunisian and 75 French families recruited via a national cooperative network focusing on familial hematological malignancies (call for proposal 2005) and the GenHem-INSERM/DGRS Franco- Tunisian project, and this network was supported by the French National Cancer Institute (INCa). The cohort consisted of 71 patients belonging to 63 familial forms of hematological malignancy (at least two cases of hematological malignancy with or without solid tumors had occurred in the patients' first, second or third degree relatives); 17 patients from 17 families with aggregated tumors where one case of hematological malignancy had occurred in the patients' first, second or third degree relatives; and 1 patient who had a multiple primitive tumor with hematological malignancy but no family history.

The genetic analysis was performed on genomic DNA extracted from peripheral blood cells obtained during complete remission. Informed consent was obtained from the patients, relevant family members (healthy relatives) or their legal guardian as required by the Helsinki Declaration. In the only case where no peripheral blood was available, tumoral DNA was prepared from paraffin embedded sections as previously described [30,31].

A control Tunisian population was recruited among healthy blood donors. Blood samples were obtained after the donors had given their informed consent.

### Analysis of perforin gene mutations

Genomic DNA was extracted from whole blood with the EZ1 DNA tissue kit (Qiagen, Hilden, Germany) in line with the manufacturer's instructions. The coding region (exon 2 and 3) and the intron-exon junctions of the perforin were amplified using standard PCR methods. The primer sequences are available upon request.

The amplified PCR products were column-purified and both strands were sequenced using the BigDye Terminator Cycle Sequencing Ready Reaction Kit v1.1 (Applied Biosystems- Foster City, USA) and loaded onto an ABI Prism 3130 sequencer (Applied biosystems). The sequence chromatograms obtained were compared with the published human *PRF1 *gene sequence (Genebank accession number M28393) using the SeqScape software program v2.5 (Applied Biosystems).

To address the issue of the occurrence of the newly observed *PRF1 *mutation c.632 C > T (p.Ala211Val) in the general population, a series of 200 Tunisian control chromosomes were examined.

### In silico analysis

To predict the effects of non synonymous SNP (nsSNP) at mRNA and amino acid levels, Align-GVGD, SIFT, ESEfinder and ESErescue bioinformatic tools, provided in the Alamut pack V2.0 http://www.interactive-biosoftware.com. Align-GVGD is a program that uses the biophysical characteristics of amino acids combined with protein multiple sequence alignments (Grantham variation and Grantham Deviation scores) to predict where amino acid substitutions fall in a spectrum ranging from enriched deleterious to enriched neutral, based on the GV an GD scores (0 to > 200). The criteria were set at GV = 0 and GD≥65, respectively [32]. The SIFT (Sorting Intolerant from Tolerant) method predicts whether an amino acid substitution affects protein function based on sequence homology and the physical properties of amino acids. Normalized probabilities of substitutions are calculated under default settings and probabilities ≤0.05 are taken to be deleterious [33]. ESEfinder [34,35] and ESErescue [36] are algorithms for analyzing several potential splicing regulatory elements.

### Cell cultures, cytotoxicity assays and perforin expression

The p.Ala211Val-*PRF1 *variant was generated using the QuickChange Protocol (Stratagene) (forward primer -5' CCAGCCCGTCTACCTCAGGC-, reverse primer -5' GCCTGAGGTAGACGGGCTGG-). The wild-type (WT) and mutant cDNA were cloned into the pIRES2-EGFP (CLONTECH) expression vector. Effector rat basophile leukaemia cells (RBL-2H3; ATCC) were cultured and transiently transfected with either wild-type or p.Ala211Val mutant variant, and populations of cells with identical mean EGFP fluorescence were FACS sorted as previously described [37]. The resultant cells were then surface labeled with anti-trinitrophenol IgE; target Jurkat T cells were first loaded with 51 Cr and then surface-labeled with trinitrobenzosulfonic acid, as previously described [37]. RBL and Jurkat T cells were subsequently mixed at various effector/target cell ratios, and the supernatants were harvested after a 4-hour period of incubation at 37°C. The 51 Cr released was measured on a gamma counter. The percentage rate of specific 51 Cr release was calculated using the formula [(experimental value-spontaneous release)/(total value-spontaneous release) ×100%]. This mutated perforin was tested 3 times at E/T ratios of 30:1, 10:1 and 2:1.

## Results

A search for *PRF1 *mutations was conducted in a total number of 89 probands identified in familial cancer clusters. The results of *PRF1 *screening are summarized in Table 1. Based on comparisons between sequences alignments, three different sequence variations were detected in three patients belonging to unrelated families: a new variant p.Ala211Val in exon 3 and two previously reported variants, p.Ala91Val in exon 2 and p.Asn252Ser in exon 3.

**Table 1:** Summary of results of *PRF1 *mutation screening on a cohort of 89 patients

Patient	Family	Geographic origin	Age at diagnosis	Diagnosis	Sex	Sequence alteration	Amino acid substitution	Family history	Predicted domain
1	F1	Tunisia	32	HL	M	c.632 C > T	p.Ala211Val	FHM without solid tumors	membrane attack complex component/perforin/complement C9
2	F1	Tunisia	36	HL	F	c.632 C > T	p.Ala211Val	FHM without solid tumors	membrane attack complex component/perforin/complement C9
3	F2	France	45	NHL	M	c.755 A > G	p.Asn252Ser	FHM with solid tumors	membrane attack complex component/perforin/complement C9
4	F3	France	43	renal cancer, Ichthyosis	F	c.272 C > T	p.Ala91Val	FHM with solid tumors	? Low homology

FHM familial hematological malignancies, HL Hodgkin lymphoma, NHL Non Hodgkin Lymphoma

**Figure 1:**
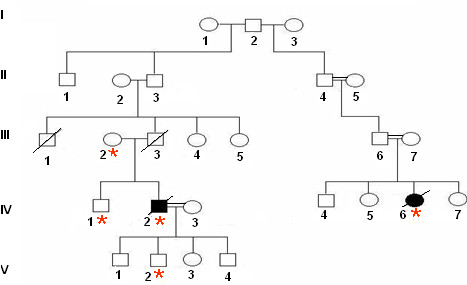
**F1 Family Pedigree**. The *PRF1 *mutation is carried by the proband's healthy mother and was also detected in his healthy brother and healthy son. The proband (Individual IV-2) had HL at the age of 32 years. His cousin (Individual IV-6) had HL at the age of 36 years. Squares stand for male and circles, for female. Open symbols represent unaffected persons, and closed symbols represent affected persons. The asterisk indicates that a germline *PRF1 *mutation was identified.

The patient from the Tunisian family F1 was a male diagnosed at the age of 32 years with nodular sclerosis Hodgkin lymphoma type II stage I. After being cured, he relapsed 5 years and a half later, showing stage III Bb Hodgkin lymphoma (HL). He developed bone metastases and died one year later. The screening of *PRF1 *led to the identification of a heterozygous missense point mutation c.632 C > T in exon 3, which transformed the Alanine in position 211 into Valine (p.Ala211Val). When permissions were available, we also screened this patient's healthy relatives (individuals III2, IV1-7 and V1-4 in Figure 1). The mutation was carried by his healthy mother and was also detected in a healthy brother and in one of his four healthy sons.

This proband had a female second cousin, who was diagnosed at the age of 36 (in 1998) with HL stage IV B b. She carried the same heterozygous missense mutation (p.Ala211Val). The treatment failed, and she relapsed twice, two and three years later, and died due to the progression of the disease. Neither her sisters nor her brother carry the mutation. Her parents' marriage was consanguineous (Figure 1).

The Align-GVGD and SIFT scores obtained on the p. Ala211Val substitution (0.35 and Class C0 (GV: 353.86, GD = 0), respectively) predicted no effect on the protein structure. The Alanine residue at position 211 was not a highly conserved amino acid (6/17 species Alamut V2.0) and the Grantham score between Alanine and Valine was 64, which is a relatively low score.

In addition, the 632C > T substitution is found to be localized 100 bp downstream of the consensus splice acceptor site. Changes in the exonic splicing enhancer pattern were observed only with ESE finder (the creation of a SRp55 motif scored 3.01 using ESEfinder). No new hexamers were predicted in this position by ESErescue. The *in silico *analysis of c.632 C > T therefore did not predict that this substitution was deleterious.

In parallel, in order to investigate the occurrence of the p.Ala211Val mutation in the population, 200 Tunisian control chromosomes were screened for the *PRF1 *gene. Interestingly, the p.Ala211Val substitution was not detected in any of the control cases.

**Figure 2:**
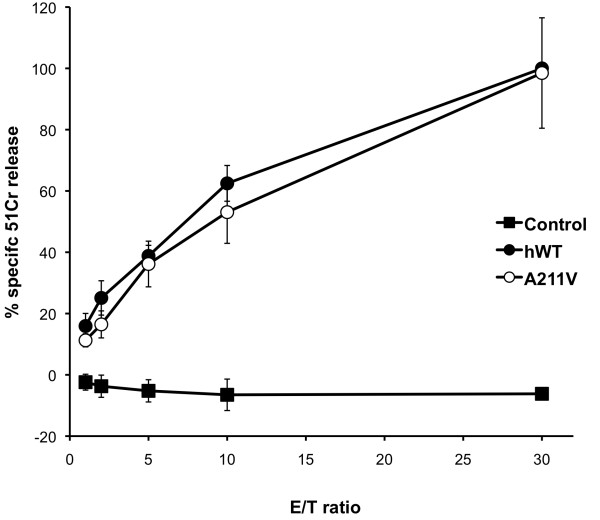
**Normal function of perforin with a valine substitution at residue 211**. The line graph shows the lytic activity of A211V perforin observed in ^51^Cr-release cytotoxicity assays using transfected RBL cells and Jurkat target cells at the E/T ratios indicated, and compares the putative perforin mutation A211V with WT human perforin and a mutated control perforin. Data shown are means ± SDs. Experiments were repeated three times.

This result combined with the particularly aggressive course of the Hodgkin lymphoma in our two patients prompted us to test whether the p.Ala211Val mutation might play a pathogenic role by performing functional test with transfection experiments. Recombinant p.Ala211Val perforin was overexpressed in RBL cells and its lytic capacity was compared with that of WT perforin. ^51^Cr-release cytotoxicity assays were performed using p.Ala211Val perforin transfected RBL cells. The p.Ala211Val substitution showed normal WT perforin and lytic activity (Figure 2).

The patient from family F2, a French male diagnosed at the age of 45 years with a non Hodgkin lymphoma (NHL), showed a heterozygous mutation c.755 G > A in exon 3 resulting in Asparagine being replaced by Serine in position 252 (p.Asn252Ser). This patient had a twin brother who died at the age of 2 from leukemia. The familial anamnesis showed signs of gastric tumors developed by the father and the grandfather at the age of 54 and 50, respectively.

The patient from French family F3, a female diagnosed at 43 years with renal cancer and ichtyosis, had a heterozygous mutation c.272 C > T in exon 2, where the Alanine in position 91 had been transformed into Valine (p.Ala91Val). Her family had suffered from multiple occurrences of cancer, as a brother and a cousin both developed chronic myeloid leukemia (CML) at the age of 34 and 37 years, respectively. The brother died of the disease.

## Discussion

The rejection of many experimental cancers by CTLs and NK cells is dependent on the pore forming protein perforin. This protein plays an important role in maintaining immune surveillance, which protects the organism from the development of lymphoproliferative disorders in vivo. Therefore, mice lacking peforin are profoundly immunodeficient and have an enhanced susceptibility to viral infection and cancer. In these experimental animal models, the increased incidence of lymphoma associated with perforin deficiency is conceivably due to a defective cytotoxic mechanism normally involved in maintaining immune homeostasis: this may favor the uncontrolled proliferation and development of premalignant lymphoid cells which may thus acquire a malignant phenotype. Stepp et al first suggested that in humans, perforin deficiency is responsible for an acute immune disregulation leading to HLH [6]. Based on mutational analyses, other authors reported the occurrence of lymphomas including B-cell, T-cell non-Hodgkin lymphoma as well as Hodgkin disease and autoimmune lymphoproliferative syndrome (ALPS) that were associated with perforin gene mutations. Bolitho et al recently established that perforin is involved in the surveillance of B cell lymphomas, as opposed to perforin loss being causative of lymphoma [20].

In the present study, the rate of occurrence of missense mutations in familial hematological malignancies was found to be 3.7%. The p.Ala211Val missense mutation detected in one Tunisian patient is a novel finding in the perforin gene: this is the first time to our knowledge that this variant has been described. The two other substitutions, p.Ala91Val and p.Asn252Ser which were identified in French patients, have been previously described and largely investigated.

The p.Asn252Ser *PRF1 *variant was first reported in a patient with FHL [6], and then identified in patient presenting with ALPS and NHL [18]. A functional analysis [38] and studies on a control population [19] have suggested that the p.Asn252Ser variant may be a benign polymorphism. p.Asn252Ser has since been proved to be a neutral *PRF1 *polymorphism.

The p.Ala91Val *PRF1 *variant has been previously reported in siblings with FHL and NHL [17,19], and in patients with Dianzani autoimmune lymphoproliferative disease [39] and aplastic anemia [40]. Here we identified this mutation in a patient with renal cancer with a family history of chronic myeloid leukemia (CML). Functional studies have shown that the levels of expression of the p.Ala91Val PRF1 protein decreased in these patients, resulting in partial loss of lytic capacity [41]. Due to an abnormal folding, the lytic activity of the mutant protein on the target cells was found to decrease 10-fold in comparison with that of the WT protein. However, the authors of the only studies available so far on this topic stated that the p.Ala91Val mutation alone did not suffice to cause the development of FHL type 2. Another event seems to be necessary to produce clinically significant effects. p.Ala91Val *PRF1 *may act as a synergistic factor with other genetic mutations predisposing patients to a larger range of cancer types such as renal cancer and CML, and these mutations may be involved in common immune surveillance and tumor escape mechanisms.

p.Ala91Val is the most common amino acid substitution identified in perforin, where the allele frequency ranges between 3% and 17%. It has long been a highly controversial case of polymorphism. It is an unusual case because it significantly affects the stability and the cytolytic activity of perforin due to the incorrect folding of the protein. It has been established that the homozygous mutation p.Ala91Val was carried by a large healthy population in whom perforin wild type function was preserved despite the presence of the p.Ala91Val substitution.

The variant p.Ala211Val was described in 5 members of one consanguineous Tunisian family; two of them were second cousins suffering from Hodgkin's Lymphoma. They died young. The probands were found to carry this germline mutation in the heterozygous state. The other family members are disease free mutation carriers. The fact that this new p.Ala211Val allele was not detected in any of the 200 control chromosomes screened suggests the pathogenicity of this mutation. However, the sequence alteration identified might also constitute a rare and hitherto undescribed polymorphism. Nonetheless, the present functional studies did not yield any evidence of altered *PRF1 *function in cells with overexpressed PRF1. This *PRF1 *variant does not predispose its carriers to hematological malignancies.

## Conclusions

The results obtained in this study show that monoallelic *PRF1 *variants alone cannot be used as prognostic factors. However, these variants might contribute to the background of genetic factors which have yet to be identified. The new p.Ala211Val seems rather to be a rare allele.

In the cohort studied here, no *PRF1 *mutations predisposing their carriers to the development of hematological malignancies were identified. It is possible, however, that a heterozygous variant of *PRF1 *might act as an inherited risk factor in addition to other genetic variations (somatic or constitutional variations in a second allele) and/or in the presence of environmental factors. Epigenetic modifications have been described in mammalian genomes which have profound effects on gene expression. It has been established, for example, that the methylation of cytosine residues at CpG dinucleotides in the promoter region favors tumor development [42]. The CpG island hypermethylation profile is specific to certain subtypes of leukemia and lymphoma [43]. However, very little is known about the epigenetic of *PRF1*. Lu et al [44] have established that DNA methylation and chromatin structure participate in the regulation of *PRF1 *expression, whereas Gao et al [45] have described how demethylating treatment can suppress NK cell cytolytic activity. It would be interesting to investigate the epigenetic of *PRF1 *to complete our study.

As immune surveillance is believed to play a major role in preventing tumor growth and/or the development of autoimmune disorders, the range of clinical manifestations associated with perforin mutations may be wider than suspected so far. Additional still undiscovered genetic defects, or possibly even environmental factors may possibly contribute along with the *PRF1 *variant to predispose carriers of this mutation to the development of familial hematological malignancies. We now intend to screen *PRF1 *in tumoral DNAs and extend our investigations to other cooperating genes. Lymphocyte mediated apoptosis involves many molecules such as Fas, Fas Ligand, Caspase 8 and Caspase10. Together with *PRF1*, these genes are excellent candidates, which we are investigating and currently analyzing in familial cancer aggregations including hematological malignancies.

The identification of predisposing genetic factors would help to develop methods of genetic counseling and genetic testing for patients with familial hematological malignancies. Inherited molecular markers make it possible to identify these high-risk individuals and the family members at risk, as well as to enroll them in appropriate screening protocols.

## Competing interests

The authors declare that they have no competing interests.

## Authors' contributions

RE carried out the molecular genetic studies, and wrote the manuscript. VB supervised sequencing data generation and *in silico *study. IV carried the functional study. HO, YBY, MAL, LH, FE, MF, PG, LD, HD, VB, CD, HZ, LF, are the clinicians who helped recruiting familial cases. MZ and SYJ helped recruiting control Tunisian population, TN and LR participated in the coordination of the study. HS and ZS conceived the study and participated in its design.

All authors read and approved the final manuscript.
